# Supplementary figures and images for: Regulation of hepatic microRNAs in response to early stage Echinococcus multilocularis egg infection in C57BL/6 mice
Source: PLoS Negl Trop Dis. 2020 May 22;14(5):e0007640. doi: 10.1371/journal.pntd.0007640 (PMC7244097; doi:10.1371/journal.pntd.0007640)

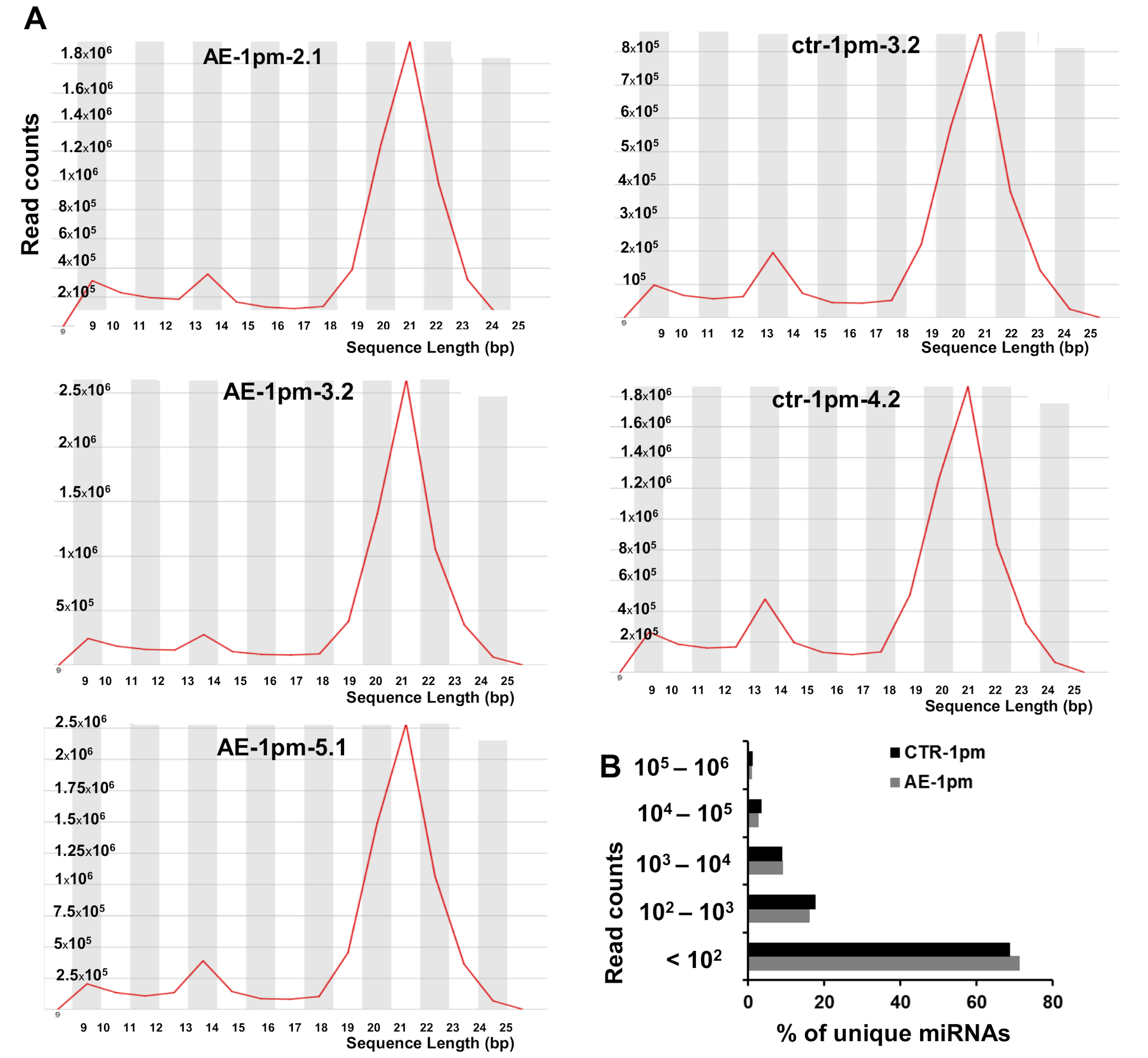

Supplement: S1 Fig — (A) Length distributions of total reads in the five small-RNA libraries. On the X-axis, reads < 10 nucleotides and reads > 25 nucleotides were discarded. The Y-axis depicts the read counts. The peak for the miRNA candidates (21 nucleotides) is centered. (B) The frequency of miRNAs that are expressed at the defined levels in each group, most of miRNAs are expressed with a read count less than 100. (TIF) [file pntd.0007640.s005.tif]

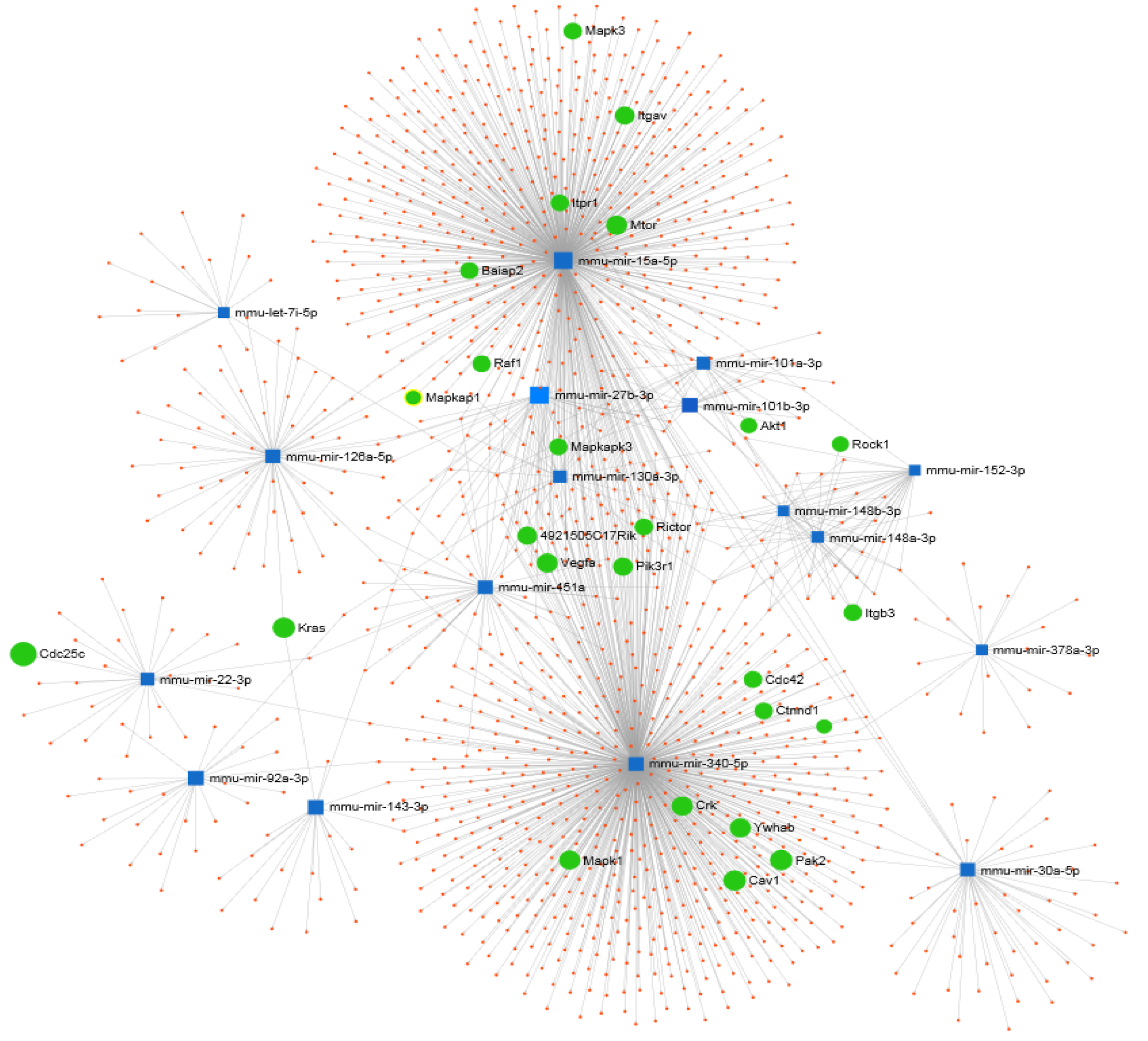

Supplement: S2 Fig — This network represents regulatory relationships between down-regulated miRNAs in alveolar echinococcosis and their target genes. Blue square: miRNAs and red dots: target genes. The 25 genes involved in VEGFA-VEGFR2 pathway are highlighted in green. The Network can be reproduced by entering the set of down-regulated miRNAs online in http://www.mirnet.ca/. (TIF) [file pntd.0007640.s006.tif]

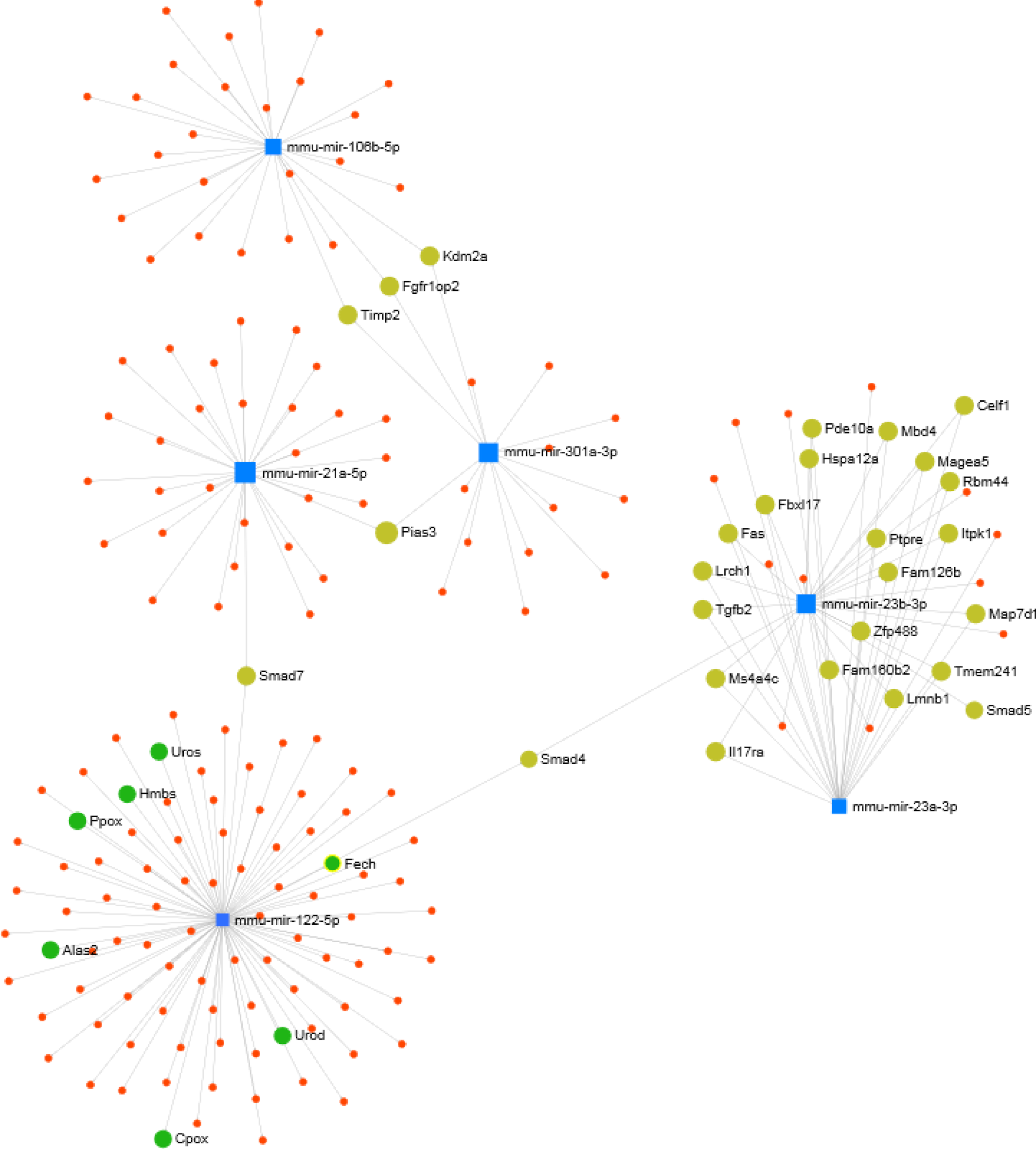

Supplement: S3 Fig — In liver of E. multilocularis-infected mice, 9 miRNAs were significantly overexpressed as compared to uninfected controls. Target genes of seven up-regulated miRNAs were predicted. This network represents regulatory relationships between up-regulated miRNAs and their target genes. Two miRNAs (mmu-mir-1839-5p and mmu-mir-28a-5p) were not connected neither to the main tree nor to each other, thus they are not presented here. Twenty-six genes are common between two microRNAs (dark yellow circles). The seven genes involved in heme biosynthesis pathway are highlighted in green. (TIF) [file pntd.0007640.s007.tif]
