# Supplementary material for: Regulation of hepatic microRNAs in response to early stage Echinococcus multilocularis egg infection in C57BL/6 mice
Source: PLoS Negl Trop Dis. 2020 May 22;14(5):e0007640. doi: 10.1371/journal.pntd.0007640 (PMC7244097; doi:10.1371/journal.pntd.0007640)
Supplement: Attachment — Submitted filename: Response_letter.docx [file pntd.0007640.s008.docx]

**Response letter:** PNTD-D-19-01183 [EMID:2740a10eb62af13a] “Regulation of Hepatic MicroRNAs in Response to Early Stage *Echinococcus multilocularis* Egg Infection in C57BL/6 mice”.

------------------

**Dear Professor Aaron R. Jex, Deputy Editor in PLOS Neglected Tropical Diseases,**

Thank you very much for the review of our manuscript entitled *"Regulation of Hepatic MicroRNAs in Response to Early Stage Echinococcus multilocularis Egg Infection in C57BL/6 mice"*.

We sincerely appreciate all valuable comments and suggestions, which helped us to improve the quality of the article. We also thank the reviewers for their constructive criticism and positive review of our paper.

We revised our manuscript and appropriate changes, as suggested by the reviewers, have been introduced in the manuscript (highlighted in yellow within the document). Our detailed responses to comments/questions made by the two Reviewers are described below in a point-to-point manner.

Our response is structured as follows: **Reviewer 1**/ 1-Methods/2-Results/3-Conclusions/4-Editorial and Data Presentation Modifications and **Reviewer 2**/1-Methods/2-Results/3-Conclusions/4- Editorial and Data Presentation Modifications. Please kindly note that we added two tables in Supplementary data and we modified two figures.

We believe that our manuscript has been considerably improved as a result of these revisions, and hope that our revised manuscript is acceptable for publication in *PLOS Neglected Tropical Diseases*.

We would like to thank you once again for considering our work and for inviting us to submit a revised manuscript. We look forward to hearing from you.

**On behalf of all the co-authors**

**Yours sincerely**

**Detailed point-by-point response to all comments/questions made by reviewers**

**Reviewer #1**

1- **Methods**

There is one detail that I found in the methods that I do not understand:

**Line 276:** "*We used cDNA samples prepared for the Stem-loop RT-qPCR for validating the set of 28 dysregulated mature miRNAs (see section above)*." How could you use these cDNA samples for qPCR of target mRNAs? They were primed using specific stem-loop primers for each miRNA...

**Response:**

We thank the reviewer for pointing out this mistake. We clarify that we used the same RNA samples for different cDNA preparations. Example: from one RNA sample (one mouse), we made (i) cDNAs for validation of most dysregulated miRNAs by Stem-loop RT-qPCR and (ii) cDNAs for assessing the relative expression of the five genes that have been identified as targets of dysregulated miRNAs.

Thus, we corrected this sentence in the revised MS: ***"* *Thus, nine cDNA preparations were synthesized on the same RNA templates which were used for validation of most dysregulated miRNAs by Stem-loop RT-qPCR (see section above)* ‶**

2- **Results**

- Yes. It would be great to have as supplementary information a complete table of miRNA expression in each sample, including also miRNAs that did not show any significant change (i.e. a full version of table 4). I would also recommend sharing the raw RNAseq data (as far as I could see, these have not been made available in a public database).

**Response:**

Following the reviewer's suggestion, we added a supplementary table (Suppl. Table 3) with all miRNAs identified in this study. The added table is cited in the results as follow “***All the 699 miRNAs, identified in this study, with the normalized read-count in each animal group, are listed in supplementary Table 3.****”*

3- **Conclusions**

- The main issue that I find lacking in the discussion is that there is no hypothesis regarding how AE changes miRNA expression in the surrounding tissues. In particular, an aspect that is not considered is that part of the differences could be related not only to differential gene expression of constituent hepatic cells, but also from the different cell composition that results from the inflammatory immune response (accumulation of macrophages, lymphocytes, etc, in the surrounding tissues).

**Response:**

- Thanks for the reviewer, we also had this reflection. However, based on our current knowledge on molecular host-*E.multilocularis* interactions and on molecular regulation of microRNA expression, we cannot directly make a detailed assumption on how AE regulates expression of hepatic miRNAs. We are convinced that stating any assumption at this point is very speculative and inappropriate due to this lack of knowledge. Concerning the heterogeneous cellular composition of the periparasitic liver tissue in AE, this was taken into account when we discussed the effect of miRNAs dysregulation on immune cells.
- In the revised version of the MS, in the beginning of the discussion, we added a paragraph to clearly state that cell composition of the analyzed liver-tissue samples from infected mice might be heterogeneous ***“The cellular composition is heterogeneous, and includes hepatocytes, stellate cells, Kupffer cells and liver endothelial cells. In addition, hepatic AE is characterized by a periparasitic infiltration of immune cells [8]. Thus our differential expression analysis of hepatic miRNAs concerns all these cell types which might be present in the isolated liver tissue samples from infected and non-infected mice.”***
- We also added a paragraph on how regulation of miRNAs expression can be achieved, and this based on literature ***“In infectious diseases, molecular mechanisms underlying the differential miRNA expression patterns remain unidentified. In addition, regulation of miRNAs expression is a complex and multilevel-process [113]. Expression of four genes involved in miRNAs biogenesis was found to be altered subsequently to liver infection with E. multilocularis [40], however this cannot explain the reported selectivity in regulating miRNAs expression. Further investigations are needed to clarify the contribution of parasites in changing expression and abundance of host miRNAs, particularly regarding the potential role of epigenetic mechanisms. In this respect, it has been reported that epigenetics, including DNA methylation and post-translational modifications of histones, regulates the expression of a notable number of miRNA genes [114]”.***
- In addition, our analysis of arm selection preferences (5p or 3p) in miRNA pairs can be considered in this frame as a regulatory mechanism of miRNAs expression. ***“ So this so-called “arm switching” phenomenon is now recognized as a miRNA post-transcriptional regulatory mechanism [122]”.***
- I am not convinced about some of the comparisons between AE and HCC - specifically, in lines 444 to 448, you compare miRNA changes between both conditions. It is true that the parasite grows like a tumour, and may affect the surrounding tissues in some similar ways. However, here you are comparing miRNA expression of tumour cells for HCC vs. miRNa expression in the surrounding tissues for AE.

**Response:**

- We respect the reviewer's opinion and we strongly think that there has been a misunderstanding. Indeed, although the paragraph as it was written in the submitted version suggests a strict comparison between AE and HCC, this was not our goal. We just report an observation on qualitative dysregulation of miRNAs in two liver diseases.
- Growth of *E. multilocularis* larvae is often compared to liver tumors (parasite versus carcinogenic liver cells), knowing that there is no study supporting this, especially with regard to molecular mechanisms responsible for the unlimited proliferation of *E. multilocularis* and cancerous liver cells. The physiological changes in liver cells from the periparasitic region have not been examined and compared to cancerous liver cells. However, as it is shown in Figure 7, 14 out of the top 30 KEGG-cellular/molecular enriched pathways were related to cancer. We cannot ignore this fact, and therefore we wanted to view our results in this direction.

In AE, the physiology of liver cells from the periparasitic area is significantly modified due to the pressure exerted by larval growth. Thus, we considered that these cells are anomalous as they are reprogrammed somehow in favor of parasite growth (condition 1).

HCC originally arises from hepatocytes that were transformed under the pressure of a given carcinogenic factor (alcohol abuse, hepatitis B/C, autoimmune diseases, etc…). Thus, we considered that cells from HCC are also anomalous liver cells (condition 2).

In both situations and regardless of the causative agent, we have abnormal livers cells and we wanted to look at similarities particularly in term of microRNA regulation.

In the revised version of the MS and to avoid any misunderstanding we changed this sentence, it is now as follow ***″*** ***On the other hand, 19 out of the identified 28 dysregulated miRNAs were reported to be also affected in hepatocellular carcinoma (HCC) which is the most common type of primary liver cancer in adults [70–78]. Nonetheless, it remains to be shown whether changes in expression levels of the 19 miRNAs are common to various liver diseases, or whether they result from common induced cellular events/mechanisms that occur in liver cancerous cells as well in periparasitic liver tissue."***

- There are several enriched pathways in figure 7 that are related to each other and probably share constituent genes. It seems likely to me that many of the genes that result in VEGFA-VEGFR2 pathway enrichment are also resulting in the enrichment of PDGF, EGFR, FGFR pathways (many of the genes indicated in Supplementary Figure 1 are largely shared between these pathways, and as far as I can see, only VEGFA is specific). In my opinion, this should be part of the description and discussion of these results, as it tones down the specific importance of VEGF. It would be good if the genes involved in pathway enrichment for each category were provided as supplementary information.

**Response:**

- We thank the reviewer for raising this point. We have taken this comment into account, in the revised version of the MS, we changed this section to the following.
- In the Results: ***‶ According to Figure 6A, Reactome pathway enrichment analysis revealed that the main enriched biological processes targeted by down-regulated miRNAs were signaling pathways activated by growth factor receptors, namely vascular endothelial growth receptor 2 (VEGFR2), fibroblast growth factor receptor (FGFR), epidermal growth factor receptor (EGFR) and platelet-derived growth factor (PDGF). Twelve genes were shared between these four signaling pathways (Supplementary Table 4). A set of 25 genes involved in “VEGFA-VEGFR2” are highlighted in the network of down-regulated miRNAs- mRNA targets (Supplementary Fig 2).″.***
- In the Discussion ***″*** ***Interestingly, our pathway enrichment analysis of down-regulated miRNAs revealed a clear enrichment of growth factor-associated signaling pathways with VEGFA/VEGFR2 being ranked the first by statistical significance."***
- As recommended by the reviewer, we also added a supplementary table (Suppl. Table 4) where genes involved in each pathway are listed. Twelve genes (Mapkap1, Cdc25c, Mapk3, 4921505C17Rik, Akt1, Itpr1, Kras, Raf1, Rictor, Pik3r1 Mtor, and Ywhab) are common between the mentioned signaling pathways (PDGF, EGFR, FGFR and VEGFR), we mentioned this in the Suppl. Table 4
- Even though I find this study superior in execution and design (as it involves samples from primary echinococcosis and greater statistical power), it would be worthwhile to include a more detailed comparison to the miRNAs found to be dysregulated by AE by Jin et al 2017 (cited by the authors) as some coincide between both studies.

**Response:**

We thank the reviewer for drawing our attention to this paper. In the revised version of the MS, we discussed our results with those reported by Jin et al. 2017, thus we added the following sentence ***"Thirteen out of the 19 down-regulated miRNAs identified in our study were also down-regulated in experimental secondary AE in DBA/2 mice at three months post-infection [40,41]. However, none of the 9 differentially up-regulated miRNAs identified herein has been shown to exhibit altered expression levels in experimental secondary AE [40,41]. This unconformity is most likely due to differences between experiments such as the route of infection, mouse strain, and infection stage."***

- Although typically mRNA target levels decrease in the presence of the targeting miRNA, the precisse relationship between miRNA and target mRNA levels can be quite variable, as most regulation occurs at a translational level. Therefore, you could mention that it is likely that the true effect on gene expression is subestimated in the qPCR experiments.

**Response:**

- We thank the reviewer for pointing out this issue and we completely agree on the partial coherence between the expression level of a given miRNA and its target mRNA. We admit that the levels of down- or up- regulation of the studied genes as seen by qPCR are not strictly proportional to the degree of change found for their specific regulatory miRNAs. MicroRNAs contribute partially to the control of gene expression at post-transcriptional level, for that reason we measured mRNA of target genes. Future investigations on AE-induced angiogenesis or changes of lipid metabolism must imperatively include comparative assessment at protein/enzymes level between infected and non-infected liver samples.
- Thereby, we included the following paragraph in the discussion ***″ However, considering that a single mRNA can be targeted by many different miRNAs [88] and that regulation of gene expression is also ensured by post-translational modifications, the relative increase of fasn and acsl1 expression levels can only be partially explained by the decrease of miR-148a and miR-15a-5p. ‶***

4- **Editorial and Data Presentation Modifications**

- **Line 24 and Line 66:** Explaining the abbreviation "*E. multilocularis*" is unnecesary (it is standard to abbreviate the genus to its inital letter after the first time that the species is named).
- **Response:** We now modified and used *E. multilocularis*
- **Line 66: cyclophyllid** -> cyclophyllidean
- **Response: we modified.**
- **Line 100**: the number of true miRNA loci is still very controversial - see for example doi: 10.1093/nar/gkz097. Perhaps mention a range of possible miRNA loci instead based on the literature.
- **Response:** We completely agree with the reviewer, the number of annotated miRNA genes in the human genome is continuously increasing, we now reformulated this sentence to the following ‶***The numbers of human miRNA-generating loci are continuously increasing and range between 2000 to 4000 [18–21][21]."***
- **Line 194:** this should probably be "libraries from five mice"
- **Response:** We thank the reviewer for pointing out our mistake, we corrected it in the new version.
- **Line 199:** "intended" -> "expected". Is the 141 bp peak the result of the addition of the 21 bp miRNA cDNA plus the adapters?

**Response:** Yes at this step (after purification), a peak at 141bp represents a tagged small RNA of ~21nt. We changed "intended" by "expected"

- **Line 238:** did you use the same primers for sno234 as in ref. 62?
- **Response:** Yes, we used the same primers described by Matoušková et al. 2014 (ref 62). To avoid misunderstanding by readers, in the revised MS, we provide full information (sequences), in Supplementary table 1.
- **Line 243:** change "5 min_25ºC" to "5 min at 25 ºC", etc.
- **Response:** we modified following the reviewer's recommendation.
- **Line 250:** add the missing parenthesis
- **Response:** we added the missing parenthesis
- **Line 270:** "Precisely" -> "Specifically"
- **Response:** we rewrote this paragraph, and we removed the word "*Precisely*"
- **Line 302:** "compromised" -> "comprised"
- **Response:** we replaced the term “compromised” by “comprised”.
- **Line 308:** you generally used miRNA as singular and plural throughout the text, but here you used miRNAs as the plural
- **Response:** we revised this issue; we went through the MS and used miRNA for single molecule and miRNAs as the plural.
- **Line 310:** "whose log2FC" -> "whose fold change"
- **Response:** it makes sense to use fold change, thus we modified it in the revised version of the MS.
- **Line 312:** "FC ≤ 0.6" should probably be "FC ≤ 0.67"
- **Response:** we corrected this
- **Line 334: "they" -> "that"**
- **Response:** we corrected it.
- **Line 344 to 348:** it would be more clear if you reversed the order of these sentences (and would result in mentioning figure 6a before figure 6b).
- **Response:** thanks for the reviewer, we re-organized the sentence and in the revised MS figure 6 is figure 5. ***"*** ***Linear regression analysis of 5p-arm selection rate in the 127 miRNA pairs showed a strong correlation (R2 = 0.9764) between the two groups of mice (Fig 5A). Particularly for nine pre-miRNA (miR-106b, miR-144, miR-16-1, miR-1981, miR-214, miR-28a, miR-335, miR-345 and miR-532) the difference in 5p- and 3p-arm expression between the two groups ranged between 10% and 33% (P < 0.01) (Fig 5B)."***
- **Line 352:** "identified for the 25 miRNAs" -> "identified for 25 miRNAs"
- **Response:** we modified this.
- **Line 366:** "alone mmu-miR-23a-3p and mmu-miR-23b-3p share 21 targets, 81% of common genes." -> I would recommend changing this to mmu-miR-23a-3p and mmu-miR-23b-3p share 21 targets, representing 81% of genes targeted by two different up-regulated miRNAs."
- **Response:** we modified this sentence accordingly.
- **Line 369:** "Immune relevant" -> perhaps change to "Genes relevant to immunity included..."
- **Response:** we changed this sentence.
- **Line 369-378:** in some cases, the same mRNA is predicted to be targeted by different miRNAs that are up and down-regulated, so the expected effect is not clear. This could be mentioned here.

**Response:** thank you for mentioning this issue. However, there is no gene among the listed ones in this paragraph which appeared to be target at the same time of an up-and down-dysregulated miRNA. We provide this more simplified to check this (***IL-1β*** 🡨 mmu-miR-122-5p ↑**/*SMA3*** 🡨 mmu-miR-27b-3p ↓and ***SMAD4*** 🡨 mmu-miR-122-5p↑**/**

***Camk2a*** 🡨 mmu-miR-340-5p ↓, mmu-miR-148a-3p↓, mmu-miR-148b-3p↓ and mmu-miR-152-3p↓**/** ***IRF7*** 🡨 mmu-miR-122-5p↑ and ***IRF8*** 🡨 mmu-miR-22-3p↓**/** ***IL-17RA*** 🡨 mmu-miR-23a/b-3p↓**/ *ICOS*** 🡨 mmu-miR-101a-3p ↓**/** ***V-CAM-1*** 🡨 mmu-miR-340-5p. ↓**/** ***ACSL***1and ***Fasn*** 🡨 mmu-miR-340-5p↓ and mmu-miR-15a-5p↓**/** ***HDAC-2***🡨 mmu-miR-340-5p ↓, ***HDAC-4*** 🡨mmu-miR-22-3p↓ and ***HDAC-9***🡨mmu-miR-340-5p ↓**/*Dnmt-1***🡨 mmu-miR-148a/b-3p ↓ and mmu-miR-152-3p↓). Horizontal arrow after the name of the gene means targeted by and vertical arrow after the name of miRNA means up or down-regulation.

We preferred to mention this fact in the discussion, we added the following paragraph "***However, one has to consider that a single mRNA can be targeted by many different miRNAs [88] and that regulation of gene expression can also mediated by post-translational modifications. Therefore the relative increase of fasn and acsl1 expression levels can only be partially explained by the decrease of miR-148a and miR-15a-5p."***

- **Line 377:** ACSL1 is not directly involved in FA biosynthesis, but in FA activation for complex lipid synthesis and catabolism.
- **Response:** we corrected this sentence.
- **Line 383** is not very well connected to the rest of the text.
- **Response: agreed,** we modified this sentence:" ***To elucidate regulatory relationships between up- or down- regulated miRNAs and the 1645 identified target genes, two interaction networks of miRNAs-mRNA targets were constructed and are shown in supplementary figures 2 and 3 (Supplementary Fig 2 and 3)."***
- **Line 397:** It could be worthwhile to mention that all mRNA related to heme biosynthesis are targeted by miR-122, an hepatic miRNA with known roles in iron homeostasis (doi: 10.1172/JCI44883)
- **Response:** Thank you for mentioning this article, we included it in the discussion. ″ ***Target genes of miR-122 are mostly enriched in heme biosynthesis and porphyrin metabolism, this may be linked to the recently reported critical role of miR-122 for regulation of systemic iron metabolism [110].″***
- **Line 401:** what do you mean here by "most significant"? They were not specifically among the enriched categories shown in Fig 7
- **Response:** Thanks for the reviewer for this remark, we agree with that. We removed the expression “most significant” and we completely changed this paragraph ***"Relative expression levels of five genes that were identified as targets of down-regulated miRNAs and are involved in angiogenesis (vegfa, mtor and hif1α) and lipid metabolism (fasn and acsl1), were further assessed by RT-qPCR in livers from AE-infected and non-infected mice (Supplementary Table 2). "***
- Line 443: "metastatis" -> "metastasis"
- **Response:** we apologize for this typing error. We revised this paragraph and we deleted this word.
- **Line 469:** actually, FASN has several domains and catalyzes all steps of FA synthesis from acetylCoA and malonylCoA.
- **Response:** we revised this paragraph and we removed this sentence.
- **Line 518:** I believe this line should be toned down; the results suggest that this phenomenon is partially mediated via downregulation of specific miRNAs.
- **Response:** we agree that we might have over interpreted our results. In the revised version of the MS, we followed the reviewer’s recommendation and changed this sentence to only suggest that the relative high mRNA levels of *vegfa*, *hif1-α* and *mtor* *in E. multilocularis* infected liver tissues might be at least partially mediated via downregulation of specific miRNAs such as mmu-miR-15a-5p, mmu-miR-126a-5p and mmu-miR-101a/b-3p.
- **Line 563:** "evaluate" -> "to evaluate"
- **Response:** according to recommendation of reviewer 2, we deleted this sentence to shorten the discussion.
- **Line 571:** "Marcella" -> "Marcela"
- **Response:** we apologize for the inconvenience and corrected this mistake.
- **In my opinion,** Figure 1 could be transferred to the Supplementary Information.
- **Response:** we agree and transferred figure 1 to the Supplementary Information.

5- **Summary and General Comments**

- This is an interesting, well executed and clearly written article in which the authors compare the miRNA transcriptome of hepatic tissues in control conditions and during early AE infection. This is a primary, hepatic infection, which is a good model for the natural infection in rodents. The authors strengthen their RNAseq results by confirming a selected number of significantly regulated miRNAs by qPCR, and they also analyze the levels of some predicted miRNA targets by qPCR.

I only have some observations and recommendations regarding the discussion of the results, and a question regarding the methods, which I already included in the previous sections. I also provided some editorial comments.

- **Response:** Thank you for the positive assessment. We revised the manuscript according to the reviewer’s comments and suggestions.

**Reviewer #2:**

1- **Methods**

- Some of the methods were not described in sufficient detail (see detailed comments below). Further evidence of how they determined levels of significance for miRNA differential transcription is required.
- **Response:** thank you for your comment. We have taken all comments/questions into consideration to improve and clarify the methodology descriptions. However, we want to explain that we have not applied novel methods in our study. When comparing the differentially expressed miRNA profiles between two groups, fold change and P-value were calculated and used to identify significantly differentially expressed miRNAs. Fold change is often used in analysis of gene expression data in microarray and RNA-Seq experiments.

2- **Results**

- Results clearly match the aims of the study. The results are presented in sufficient detail. The link between their results in fatty acid biosynthesis were not very clear. Perhaps more information is required (pathway genes listed? Synonyms made clear for a broad audience?)There are a lot of tables and figures presented in the main body of the manuscript. Some consolidation of figures and moving quality control results to the supplementary material is recommended. Additional comments about the results are listed below.
- **Response:** Thanks for these comments. The manuscript has been modified taking into account all these suggestions.
- We made a clear explanation concerning our choice to assess the relative mRNAs level of two enzymes involved in fatty acid biosynthesis. In fact, both *fasn* and *acsl1* are validated targets of mmu-miR-15a-5p (down-regulated in our study).
- We added a supplementary table (Suppl.Table 4) with a list of genes involved in each pathway.
- Only tables and figures directly related to the results section are maintained in the main body, the others were moved to supplementary data.

3- **Conclusions**

- The discussion is quite long and the authors have made a number of conclusions from this data set. I would recommend they be more cautious considering the small changes in transcription that were observed (particularly the mRNA validation).
- **Response:** thank you for this comment. The discussion section has been shortened. In the revised version of the MS, we re-interpreted our findings on mRNA expression of the five genes more cautiously, with less overinterpretation.

4- **Editorial and Data Presentation Modifications**

- **Lines 54 to 56.** Sentence unclear, please check grammar
- **Response:** we reformulated this sentence in the updated version of the MS.
- **Lines 84.** Sentence is unclear. Please check for grammatical errors**.**
- **Response:** we rewrote this sentence. There was a typo error. ***“Currently, there is no alternative to benzimidazole-based chemotherapy for patients suffering from benzimidazole intolerance [11].”***
- **Lines 100 to 101.** Sentence is unclear. Perhaps it needs to be clear what a gene is first (coding and non-coding)?
- **Response:** we rewrote this sentence. ***"*** ***Based on computational predictions it is estimated that more than 60% of all human protein-coding genes harbor at least one conserved miRNA-binding site "***
- **Lines 141 to 142.** Unclear if you are referring to the miRNA or the mRNA that they target.
- **Response:** we rewrote this sentence, ***"Furthermore, we used infected and non-infected livers tissue samples to comparatively measure the relative mRNA levels of five genes that have been identified as targets of dysregulated miRNAs."***
- **Line 184.** Can you include the company and product information for the DNase I?
- **Response:** we added the company’s name and the catalog number for DNase I. ***DNase I (Thermo Fisher Scientific, Cat: EN0521)***
- **Lines 201 to 202.** Methods is unclear here.

**Response:** we clarified this sentence. ***In a last step before the deep sequencing, the quality and concentration of each final library (the eluted 141 bp band) were submitted to PicoGreen analysis (Thermo Fisher Scientific, Cat:P7589).***

- **Lines 202 to 204.** This statement is unclear. How long were the reads sequenced? is 50 Mio 50 million. What is past filter?
- **Response:** We mentioned in text that we used high-output v2 Kit for a 75-cycles sequencing run. Yes we used mio as abbreviation for millions, we modified and used the full word. Sequencing instruments from Illumina Company execute an internal quality filtering procedure termed chastity filter. The filter aims to remove the least reliable data from the analysis results, often derived from overlapping cluster. Reads that pass this filter are called PF for pass-filter.
- **Lines 218 to 219.** Not clear what they mean by valid here? Please revise and make it clearer.
- **Response:** by using the term “valid” we meant those hits with a significant match. In the revised version of the MS, we completely reformulate this sentence. "***Therefore for the blast against Rfam, we choose an Expect value (E) threshold of less than 1; the E- value criteria describe the number of expected hits occurring by chance. According to this, we considered hits with an E-value ranged between 0 and 1 as significant match."***
- **More detail is required** for the headers/description of Tables. Table 3 is difficult to understand with the information provided in the headers
- **Response:** we modified header of the Table 3 in order to make it easier to understand. We also provided explanation for columns.
- **Lines 403 to 407**. This reads like methods to me
- **Response:** we revised this section and moved this part to the MM section.
- **Lines 56.** You mention pathways but have not introduced the pathways you are referring to.
- **Response:** we changed the paragraph content since it was also recommended by reviewer 1.
- **Line 407.** Levels... typo
- **Response:** corrected.
- **Lines 423 to 424.** Sentence is unclear
- **Response:** we reformulated this sentence in such a way that it is comprehensible without ambiguity.

**Body of the manuscript**

- **Beginning of the abstract reads** more like a summary of the materials and methods. I don't think the methods needs to be included in the abstract with this much detail. Summary introduces hypotheses generated from the results that were not mentioned in the abstract. Abstract needs to be revised to include more results and less materials and methods.
- **Response:** We rewrote the abstract. We have shrunk the methodology to essential information about the infection model, infection stage and we mentioned techniques we have used. We cannot completely delete this paragraph, since according to Submission Guidelines of Plos NTD, the abstract should mention the techniques used without going into methodological detail. In the last part “Conclusions/Significance” we gave questions raised by the obtained results.
- **Introduction is very long**. Some information is not required. The role of miRNA is very well established now. Perhaps the role of miRNA in parasite infections is all that needs to be introduced (i.e. human miRNA and their role is already well established and needs no introduction here).
- **Response:** Our concept in writing the introduction was to provide useful and sufficient background on miRNAs, because we target different categories of readers: those working with AE infection or other infectious/non-infectious diseases and those working on miRNAs. It is not assumed that all readers are familiar with miRNAs biology, for those who have large knowledge about miRNAs, they can simply skip this paragraph.
- **Lines 157 to 165. I** assume this method is well established and has been published previously? Perhaps there is no need to detail the methods if it has already been published?
- **Response:** the intestinal scraping technique (IST) was initially established by Deplazes & Eckert (1996). In our manuscript and in the MM section, we adapted this protocol to our Lab conditions and we described briefly how we did it. We also mentioned this reference in the revised version. We decided to keep this paragraph because we think it is useful for the readers to know how the material was obtained.
- **Figures 1A** could be presented as one graph. Each line would be a different library. Further consolidation of the figures in this paper would allow them to be printed larger and improve the readability. This figure could also be removed to Supplementary files.
- **Response:** thank you for this comment. First we confirm that we checked all figures for their quality by PACE, a tool provided by PLOS. PACE assesses whether submitted figures fulfill technical requirements for PLOS journals. In addition, we cannot merge these graphs for the following reasons: **(i)** these curves were generated by the computer software and we do not want to interfere with that **(ii)** if we include all 5 curves in a single graph, it will be a complete overlap which in turn will not allow to differentiate between libraries. Following the recommendation of reviewer 1 in this regard, we transferred figure 1 to the Supplementary Information.
- **Grey labelling of the different miRNA in Fig 2B** is not described in the Figure legend. What is the significant of the different colors? Common and unique to the different groups? Some additional description in the Figure legend would be required.
- **Response:** thank you for this comment. In the revised version of the MS, we revised legend for figure 2 (Figure 1 in the revised MS) and we clarified the indication of the grey and black labeling of some miRNAs. Grey labelled miRNAs are only present in the top 10 most abundant miRNAs in the infected group, whereas black labelled miRNAs are only present in the top 10 most abundant miRNAs in the control group.
- **Fig 6B description is before 6A.** Panels need to be swapped on Fig 6.
- **Response:** as it was also notified by reviewer 1, in the revised MS Figure 5A appears before 5B.
- **Discussion section is quite long.** Conclusion paragraphs could be significantly reduced. For example, there is quite a bit of talk about biomarkers and this is not required here, simply mentioned at the end of the last sentence perhaps?

-	**Response:** according to the Reviewer’s comments discussion & conclusion have been shortened.

- **Tables 1 and 2** could be moved to supplementary tables. Table 3 requires more detail to improve understanding of each column.
- **Response:** we moved Tables 1 and 2 to the supplementary information. We provided more details for table 3 (see below)

5- **Summary and General Comments**

- When annotating the miRNA using BLAST: Would you not be looking for a perfect match to these nucleotide sequences? Please justify this two step process of annotating putative miRNA. Conducting BLAST searches against miRBASE and Rfam may not be the most reliable method of annotating your miRNA. For example, Rfam would recommend using Infernal (HMM-based searching) to annotate small RNA using the Rfam database.
- **Response:** thank you for mentioning this point. In fact, we want to clarify that blast against the Rfam collection of RNA families is not the main step in our methodogy. By using this database, we did not need to annotate nor intended to (this was exploratory to see what else might be there in addition to what miRBase provided). In reality, Rfam section brings no real value as the miRNA stem from miRBase it comes already fully annotated for the mm10 reference genome. For this reason we decided to move this paragraph as it is the last step in the analysis (verification step). We believe that this re-organization in the text will avoid any misunderstanding by readers. "***Lastly and in an exploratory step to check for what else might be present in our data in addition to what the miRBase provided, we performed a blast against the Rfam collection of RNA families (v. 12.3) [57]. This blast concerned only sequences that did not show a hit with an e-value of at least 1e-6 or smaller during the blast against the miRBase. Therefore for the blast against Rfam , we choose an Expect value (E) threshold of less than 1; the E- value criteria describe the number of expected hits occurring by chance. According to this, we considered hits with an E-value ranged between 0 and 1 as significant matches."***
- **Lines 219 to 221.** The link from small RNA clustering, annotation to mapping is unclear. When mapping to the genome, how were the locations of your miRNA determined. Usually the premature miRNA would be used for positioning the miRNA within the genome. In this case, were you mapping to the mature, the star or the mature, star and hinge all at the same time? More information is required here.

**Response:** We used the STAR aligner in conjunction with the mm10 reference genome assembly to map the miRNA reads. Uniquely mapped reads were then counted using the software htseq-count and the miRBase gff file on mm10 miRNAs which provided information about the position of known mature miRNA along the mm10 reference genome.

- **Line 225.** If this is the case, then how did you annotate miRNA that were not a match to miRNA encoded in the reference genome? (see Lines 217 to 218)
- **Response:** Hereby, we clarify again that we did not need to annotate miRNAs nor intended to. In the revised MS, we included Blast in Rfam as exploratory analysis on what else there could be in the data beside the known miRNAs (as of miRBase).
- **Lines 226 to 227.** Were the distributions of miRNA transcription normally distributed? Can often be tricky when performing pairwise comparisons tests using skewed data (often observed with miRNA data). Also, what settings were used? fold change, and multiple testing correction q value? I assume multiple test correction was performed as part of the DESeq2 pipeline. Overall, more information should be included about the steps taken to determine the differentially transcribed miRNA. Currently it is too difficult to work out what has been done.
- **Response:** Yes multiple testing p-value adjustment was done using the Benjamini-Hochberg method. We were analyzing the data following the methods implemented in DESeq2: https://www.ncbi.nlm.nih.gov/pmc/articles/PMC4302049/ and recommendations given here: <https://bioconductor.org/packages/release/bioc/vignettes/DESeq2/inst/doc/DESeq2.html>. Concerning, the normalization step, we used DESeq2 as it is mentioned in the text and also we provided references. Briefly, DESeq2 performs an internal normalization where geometric mean is calculated for each gene across all samples. The counts for a gene in each sample is then divided by this mean. The median of these ratios in a sample is the size factor for that sample. This procedure corrects for library size and RNA composition bias, which can arise for example when only a small number of genes are very highly expressed in one experiment condition but not in the other (https://chipster.csc.fi/manual/deseq2.html). For more detailed explanation, the reviewer could consult this article (M Love, W Huber and S Anders: Moderated estimation of fold change and dispersion for RNA-Seq data with DESeq2. Genome Biol. 2014 15:550).
- **Could the authors** also comment on the use of the DESEQ2 pipeline when your number of replicates is 2 in the control group?

**Response:** DESeq2 requires a minimum of two samples to form a replicate group for the statistics to work.

- **Lines 229 to 230.** Not sure what you mean about the 2D here. Do you mean that you chose to only display 2 dimensions? Presumably the 2 dimensions that accounted for the most variation?
- **Response:** in the revised MS we have developed this paragraph to better explain the meaning of 2D which refers to number of compounds. ***"We applied principal component analysis in two dimensions (2D-PCA) [62] to check whether control mice (ctr-1pm) could be distinguished based on their miRNAs expression profiles from those experimentally infected with E. multilocularis eggs. The PCA statistical approach is applied to complex data sets and aims to reduce the dimensionality of the data matrix while simultaneously retaining the maximum amount of variance. The term 2D refers to the number of used principal components: the first component, PC 1, represents the direction of the highest variance of the data and PC 2 points into the directions of the highest of the remaining variance orthogonal to the first component. "***
- **Line 277.** Unclear which 28 mature miRNAs are being referred to here. There are less than 28 primer sets listed in Table 1.
- **Response:** we note that there was a confusion in this sentence. We revised the whole paragraph as followed: ***" Thus, nine cDNA preparations were synthesized on the same RNA templates which were used for validation of most dysregulated miRNAs by Stem-loop RT-qPCR (see section above)"***
- **Lines 293 to 294.** So most miRNA had mapped reads aligned at a depth of 10 to 100? Is this quite low or normal for mouse studies? Unclear why you then chose to use a depth cutoff of 1000 (see Line 312). You are removing most of your data. Particularly as it was in combination with such a low fold change coverage (1.5 fold change)? Further justification for your methods is required.
- **Response:** yes, the fact that most miRNAs identified in our study were of a read count <100, is very normal and it was reported in many studies. For example, in the study of Jin et al 2017, on murine hepatic miRNAs subsequent to secondary experimental AE, of _~_74% of identified miRNAs were detected with read counts of less than 100 (Table S1).

The cutoff of 1000 read counts is often used in such analysis, we are interested in abundant miRNAs being present in liver. MiRNAs with low read counts (<100) might not be specific, or are present just by chance in the analyzed sample.

For example if we consider a given miRNA with a read count of 2 in one group and 12 in the other group: theoretically we are in the case of one dysregulated miRNA with fold change of 6, but in reality this does not make sense.

Concerning the fold change, classically for mRNA-Seq, a 2-fold-change cutoff is used, but for miRNAs, in literature, a 1.5-fold change is often used.

In the revised version we added a Table (Suppl. Table 3) with all miRNAs identified in this study, so that the readers can access the complete data from our study.

- **In Figure 2A**, you refer to 124 AE-specific miRNAs with read counts < 100. Do you mean > 100?
- **Response:** There is no mistake, we meant read counts < 100. In the revised version of the MS, we have mentioned this in the text. ***"*** ***Specific miRNAs to AE-1pm (124 miRNAs) or to ctr-1pm (24 miRNAs) libraries were all present with read counts below 100."***
- **Methods for Figure 3B** are not included in the materials and methods section. Unclear what values are used in the legend on Fig 3B. More information required.
- **Response:** we now added a paragraph in MM section to explain this figure 3B: ***"In parallel to 2D-PCA, we also applied the hierarchical clustering method [63] for analyzing hepatic miRNA transcriptomes data from infected and non-infected mice. Hierarchical clustering consists in building clusters of libraries with similar patterns of expression. Results are displayed as a tree diagram (dendrogram)."***
- **Lines 312 to 317.** How can let 7 miRNA be up and down regulated? star and mature miRNA? More detail on let 7 genes may need to be described. Is this an example of arm swapping?
- **Response:** let-7 is a large family of microRNAs with at least 10 identified members including let-7a, let-7b, let-7c, let-7d, let-7e, let-7f, let-7g, let-7i. In our study, let-7i-5p was down-regulated and let-7d-3p was up-regulated, they are two different miRNAs.

mmu-let-7i-5p: ugagguaguaguuugugcuguu

mmu-let-7d-3p: cuauacgaccugcugccuuucu

- **Fig 6B.** Unclear what the error bars represent in this panel. It is a stacked bar plot which equals 100%
- **Response:** Thanks for the reviewer for this question. We revised this figure, now it is ***Figure 5B***, ***"Black error bars represent standard deviation in percent of the 3p arm, and white bars represent standard deviation in percent of the 5p arm."***
- **Lines 383** Production of this result is not described in the materials and methods section. I suggest they be deleted. There is also no description of this result.
- **Response:** Thanks for the reviewer for this question. In the revised version of the MS, we added a paragraph in the MM section to describe how we can reproduce theses networks.

***"Furthermore, miRNet allowed us to visualize miRNA-target interactions in a network context. Thus, analysis for pathway enrichment and displaying of interaction networks can be reproduced at any time using miRNet, a web-based tool freely available at http://www. www.mirnet.ca. For that, the list of down- or up- regulated miRNAs is used as input; selected parameters are M. musculus (mouse) for organism, miRBase ID for the ID type and genes for target type. When choosing KEGG or Reactome database, a table containing the list of pathways and the number of involved genes will be shown. By clicking on a given pathway, the names of involved genes will be displayed."***

- **Lines 401 to 402.** Here you mention that fatty acid synthesis was a significantly afffected pathway but earlier you state that it is angiogenesis and axon guidance. I couldn't see the link between axon guidance and fatty acid synthesis. More information is required here.
- **Response:** we agree with this argument, this sentence is confusing. In the revised version of the MS, we modified this to ***"Relative expression levels of five genes that have been identified as targets of down-regulated miRNAs and are involved in angiogenesis (vegfa, mtor and hif1α) and lipid metabolism (fasn and acsl1), were further assessed by RT-qPCR in livers from AE-infected and non-infected mice (Supplementary table 2)."***
- **Lines 424 to 426.** Without comparing miRNA profiles between human AE and other liver diseases it is difficult to see how you this statement is supported by your research.
- **Response:** Our global idea here is to mention that the miRNA transcriptome is often affected in liver diseases, and AE as infectious disease of the liver causes also changes in hepatic miRNAs. We are not stating that miRNAs expression in AE is altered by the same way like in other liver diseases. In the revised version of the MS, we changed this sentence to ***"In total, we identified 28 miRNAs that were differentially expressed between infected and non-infected mice at 1 month post-infection. In general, alterations in the microRNA transcriptome have been reported for a wide range of infectious- and non-infectious liver diseases [69]."***
- **Lines 516 to 518.** Discussion concludes that there is strong evidence. Levels of transcriptional change are quite low for many of the genes you tested. I would be cautious in drawing too many conclusions from the data. At least, change the wording to make it clear that you are developing hypotheses that require further testing.
- **Response:** we have revised our discussion and conclusion, and we believed that in the revised version, we avoided overinterpretation of the results, in particular concerning angiogenesis and lipid metabolism.
