# Supplementary material for: Regulation of hepatic microRNAs in response to early stage Echinococcus multilocularis egg infection in C57BL/6 mice
Source: PLoS Negl Trop Dis. 2020 May 22;14(5):e0007640. doi: 10.1371/journal.pntd.0007640 (PMC7244097; doi:10.1371/journal.pntd.0007640)
Supplement: Attachment — Submitted filename: response_letterR2.docx [file pntd.0007640.s009.docx]

**Response letter:** (PNTD-D-19-01183R1) - [EMID:e25cb2def5f48bf4] “Regulation of Hepatic MicroRNAs in Response to Early Stage *Echinococcus multilocularis* Egg Infection in C57BL/6 mice”.

------------------

**Dear Professor Gabriel Rinaldi, Associate Editor in PLOS Neglected Tropical Diseases,**

Thank you very much for the review of our modified (after the first revision) manuscript entitled *"Regulation of Hepatic MicroRNAs in Response to Early Stage Echinococcus multilocularis Egg Infection in C57BL/6 mice"*.

We sincerely thank reviewers for revising our manuscript for a second time. We appreciate the effort they made to improve our manuscript and are grateful for their insightful comments during the first and second revision.

We revised our manuscript and made all editorial modifications, as suggested by the referees. Our detailed responses to comments/questions made by the two Reviewers are described below in a point-to-point manner.

Our response is structured as follows: 1-Methods (**Reviewer 1 and 2)** / 2-Results (**Reviewer 1 and 2** /3-Conclusions (**Reviewer 1 and 2)** /4-Editorial and Data Presentation Modifications (**Reviewer 1 and 2).**

We would like to thank you once again for considering our work and for inviting us to submit a revised manuscript.

**On behalf of all the co-authors**

**Yours sincerely**

**Bruno Gottstein**

**Detailed point-by-point response to all comments/questions made by reviewers**

1- **Methods**

- **Reviewer #1** (No Response)
- **Reviewer #2:** Satisfied with most changes made in R1. Response to my comments on the experimental design and use of statistics was not complete, but the design is robust enough for the revised conclusions in R1

**Response:** We thank the reviewer 1 and 2, in particular for all constructive suggestions concerning our methodology, made during the first revision.

2- **Results**

- **Reviewer #1** (No Response)
- **Reviewer #2:** Satisfied with the changes made in R1

**Response:** We thank the reviewer 1 and 2.

3- **Conclusions**

- **Reviewer #1** (No Response)
- **Reviewer #2:** Satisfied with the changes made in R1

4- **Editorial and Data Presentation Modifications**

- **Reviewer #1**
- Line 247 - some corrections were missing.

**Response:** We now modified and used "at" instead of "_"

- Line 308-310 - you mention first 45 ctr-1pm specific miRNAs, but then 24 ctr-1pm specific miRNAs.

**Response:** we apologize for the inconvenience and corrected this mistake; we now modified and wrote the right number 45.

- Line 375 and line 541 - you mention 8 up-regulated miRNAs, but they are actually 9, as mentioned elsewhere

**Response:** We confirm that the number of up-regulated miRNAs is nine and the number of down-regulated miRNAs is 19. In the results section, the paragraph (linie362 – linie 399) with the following subtitle "Prediction of the target genes of dysregulated host miRNAs" concerns dysregulated miRNAs with predicted/identified target genes.

We mention in the beginning of this paragraph (364-366), that among the 28-dysregulated miRNAs we found targets only for 25 miRNAs. No target genes have been found for mmu-miR-148a-5p, mmu-miR-30a-3p and mmu-let-7d-3p. Both mmu-miR-148a-5p, mmu-miR-30a-3p are from the cluster of down-regulated miRNAs and mmu-let-7d-3p is one of the nine down-regulated miRNAs.

In linie 370: "For the 17 down-regulated miRNAs, a set of 1426 target genes were found".

In linie 375: "On the other hand, for the eight up-regulated miRNAs, 219 target genes were identified…"

In fact, in linie 370 and 375, 17 and 8 are the number of down- and up- regulated miRNAs for which we could identify their predicted/validated target genes.

- Line 542 -"cautiously indictaes" - I would remove "cautiously";change indictaes for indicates

**Response:** We thank the reviewer for the suggestion. In the revised version we removed "cautiously", and changed "indictaes" for "indicates"

- S Fig 1 - the x axis says "lenght" instead of "length"

**Response:** We thank the reviewer for pointing out this error. We prepared a new version of S Fig 1 with a correct labeling of the x-axis.

- **Reviewer #2:** Satisfied with the changes made in R1

5- **Summary and General Comments**

- **Reviewer #1:**

The authors have satisfactorily modified the manuscript and replied to the reviewers. I have only included a few additional editorial comments above.

The only additional thing that I have not found in the manuscript is where the original sequencing data was deposited.

**Response:** We thank the reviewer for the constructive work he/she made to improve our manuscript.

All sequencing raw data from the five libraries have been deposited in the Gene Expression Omnibus (http://www.ncbi. nlm.nih.gov/geo/) under the following accession numbers : for uninfected control mice(GSM4367926 and GSM4367927) and for *E. multilocularis*- infected mice (GSM4367928, GSM4367929 and GSM4367930) (submitted by G. Boubaker). In the revised version of the MS, we have mentioned these reference numbers.

- **Reviewer #2:**

Thank you for addressing the majority of my comments

**Response:** We also thank reviewer 2 for raising important questions. We sincerely appreciate the time she/he has invested to improve our paper.
